# Perception of Parents Towards COVID-19 Vaccine for Children in Saudi Population

**DOI:** 10.7759/cureus.18342

**Published:** 2021-09-28

**Authors:** Bader A Altulaihi, Talal Alaboodi, Khalid G Alharbi, Mohammed S Alajmi, Hamad Alkanhal, Ahmed Alshehri

**Affiliations:** 1 Family Medicine, Ministry of National Guard-Health Affairs, King Abdullah International Medical Research Center, Riyadh, SAU; 2 Family Medicine, King Saud Bin Abdulaziz University for Health Sciences College of Medicine, Riyadh, SAU; 3 Medicine, King Saud Bin Abdulaziz University for Health Sciences College of Medicine, Riyadh, SAU

**Keywords:** acceptability, parents, perception, vaccine, covid-19

## Abstract

Background

Coronavirus disease 2019 (COVID-19) is a contagious disease that is caused by severe acute respiratory coronavirus 2 (SARS-CoV-2). With the rapid spread of this pandemic, vaccination has been a breakthrough solution. At the time of conducting the study, COVID-19 vaccines were only approved for adults 18 years and older. Therefore, the aim of the study was to assess the parents’ likelihood of vaccinating their children once the recommendation for pediatric vaccination is established.

Methods

This was a cross-sectional study in which a self-administered survey was distributed to all parents visiting National Guard primary healthcare centers in Riyadh, Saudi Arabia. The questionnaires were distributed to parents attending primary care clinics. Data collected in the questionnaire include demographics (gender, marital status, educational level, and age), questions assessing parental perception towards the COVID-19 vaccine, and willingness to offer the vaccine to their children.

Results

A total of 333 respondents completed the survey with a response rate of 83.3%. Half of the participants were males and the other half were females with the majority (45.6%) aged between 31 and 40 years old. In terms of parental acceptability of vaccinating their children against COVID-19, 53.7% of the parents were willing to vaccinate their children as opposed to 27% who were reluctant to do so. Of those who refused, 97.5% and 96.6% cited lack of information and evidence, respectively, as the most common reasons for not accepting COVID-19 vaccine. We have found that age of the parents, especially those 31-40 years old, age of their children, especially 4-12 years old, and previous acceptance of the seasonal influenza vaccine were significantly associated with higher parental acceptability of COVID-19 vaccine. In contrast, gender, marital status and educational level were not statistically significant factors.

Conclusion

As COVID-19 spread globally and made people's lives in danger, vaccination became a highly important measure to halt the spread of the disease. Parents are now given the choice of protecting their beloved children from COVID-19 infection and its possible complications. Based on our findings, we noticed that majority of parents are going to vaccinate their children. In addition, some certain age groups of parents and children were significantly associated with decreased vaccine hesitancy to take the COVID-19 vaccine.

## Introduction

Coronavirus disease 2019 (COVID-19) is a contagious disease that is caused by severe acute respiratory coronavirus 2 (SARS-CoV-2). Since the start of the COVID-19 pandemic until August 18, 2021, over 200 million confirmed cases with over four million deaths [1]. The disease began in late 2019, and in 2020 many countries were already implementing curfews and social distancing. The Kingdom of Saudi Arabia was one of the earliest countries to take precautions against the disease by implementing curfew and even penalty on people who did not follow the safety guidelines [2]. Yet, the disease caused severe harm to the population [3]. The vaccine was finally introduced in late 2020, providing an elemental step into halting spread of the disease. However, many people feel reluctant about taking the vaccine in various countries all over the world [4].

People around the world might be hesitant to take the COVID-19 vaccine for various reasons. As different COVID-19 vaccines were used and distributed around the world, the rumors across social media started to spread. Rumors about the safety and potential side effects are making people hesitant to take the vaccine [5]. In addition, interpersonal communication could lead to hesitancy due to sharing of these rumors or conspiracy theories [5]. 

The ratio of population covered by the vaccination is high in Saudi Arabia as compared to other countries, with total vaccine doses given reaching 31.7 million [6]. Increased public acceptance regarding COVID-19 vaccine could have a decisive role in the successful control of the pandemic. Different studies showed an increased acceptance rate among the public which explains the high total doses of COVID-19 vaccine given in Saudi Arabia [7].

To our knowledge, no studies in Saudi Arabia have investigated parental acceptance of COVID-19 vaccination for their children. Therefore, this study aimed to explore the view of parents in Saudi Arabia on the acceptability of COVID-19 vaccine for their children as the vaccine becomes approved for those less than 18 years of age.

## Materials and methods

This was a cross-sectional study in which a self-administered survey was distributed to all parents visiting National Guard primary healthcare centers. The questionnaire used was originally developed by the JC School of Public Health and Primary Care and the Chinese University of Hong Kong. The questionnaire was edited, translated into Arabic, and then validated using a pilot study on 10 parents/caregivers to assess clarity and readability of the questionnaire. After validation, the questionnaires were printed and distributed to parents attending primary care clinics. Each participant had a unique serial number. The cover page of the questionnaire included a short introduction regarding the objectives, procedures, the voluntary nature of participation, declarations of confidentiality and anonymity. Data collected in the questionnaire include demographics (gender, marital status, educational level, and age), questions assessing parents’ perception towards COVID-19 vaccine, and willingness to offer the vaccine to their children. The inclusion criteria were all parents visiting National Guard primary healthcare centers in Riyadh, Saudi Arabia. Exclusion criteria for this study include any duplicates of the survey. The sample size was calculated to be 400 using Raosoft website with a population of 150,000 parents visiting National Guards primary health care clinics. Our margin of error was set at 5% and the confidence interval was 95%. Convenience sampling, in which all possible individuals were invited to participate in the study, was used.

## Results

This study involved 333 parents to evaluate their perception towards COVID-19 vaccination for their children. Table 1 presents the socio-demographic characteristics of the parents. The most common age group was 31-40 years old (45.6%) with slightly more males (50.2%) and most of them were married (95.8%). With regards to parents’ education, nearly two-thirds (63.1%) were college degrees or higher and 55.6% were not working. Furthermore, 35.1% had a previous history of seasonal influenza vaccination within one year. The proportion of parents who had a family history of COVID-19 was 52.6%. In addition, 40.2% of the subjects had a child whose age ranged from 7 to 12 years old. 

**Table 1:** Sociodemographic characteristics of the parents (n=333).

Study variables	N (%)
Age group	
18 – 30 years	97 (29.1%)
31 – 40 years	152 (45.6%)
>40 years	84 (25.2%)
Gender	
Male	167 (50.2%)
Female	166 (49.8%)
Marital status	
Married	319 (95.8%)
Divorced	14 (04.2%)
Educational level	
High school or below	123 (36.9%)
College degree or higher	210 (63.1%)
Type of work	
Frontline worker	74 (22.2%)
Management staff	74 (22.2%)
Not working	185 (55.6%)
History of seasonal influenza vaccination	
No	164 (49.2%)
Yes, within one year	117 (35.1%)
Yes, beyond one year ago	52 (15.6%)
Family history of COVID-19	
Yes	175 (52.6%)
No	158 (47.4%)
Child age	
0 – 3 years	70 (21.0%)
4 – 6 years	68 (20.4%)
7 – 12 years	134 (40.2%)
13 – 17 years	61 (18.3%)

**Figure 1:**
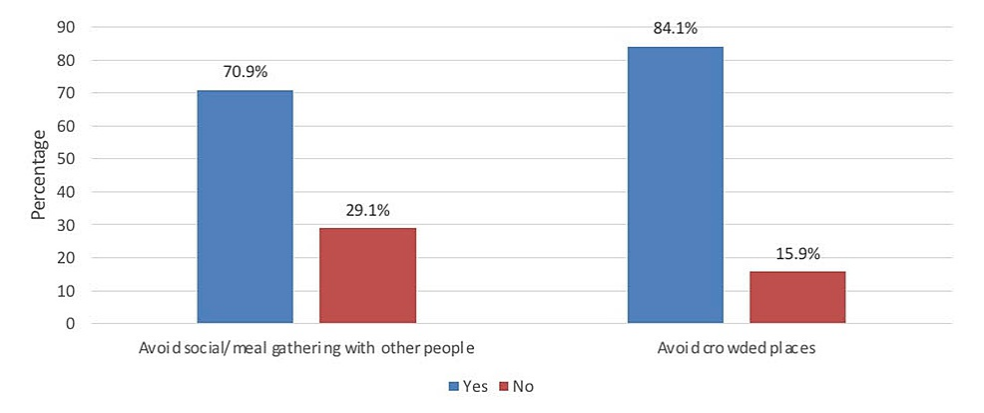
Self-reported precautions on social gatherings and crowded places.

Figure 1 shows self-reported precautions on social gatherings and crowded places. It can be observed that 84.2% and 70.9%, respectively, were avoiding crowded places and social/meal gatherings with other people. 

**Figure 2:**
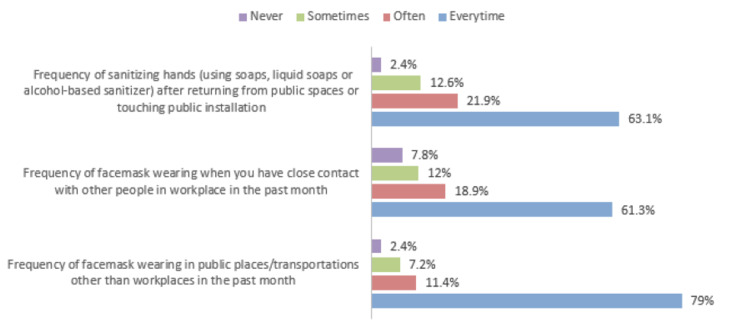
Personal COVID-19 preventive measures in the past month.

Figure 2 illustrates the personal COVID-19 preventive measures in the past month. It was shown that 63.1% of parents were frequently practicing hand sanitization after returning from public spaces or touching public installations. Also, 61.3% were consistently practicing wearing a facemask after close contact with other people within the workplace and 79% were consistently wearing a facemask in public places/transportations in the past month. 

**Figure 3:**
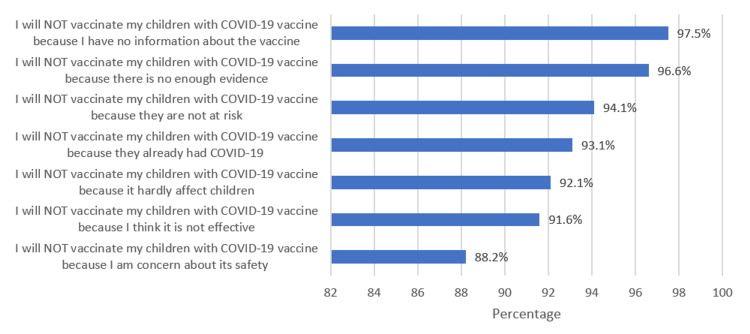
Attitude of parents who were against COVID-19 vaccination.

In Figure 3, the most common reasons for not vaccinating their children, as reported by the parents, was lack of information about COVID-19 vaccine (97.5%) followed by lack of evidence about the vaccine (96.6%). The third most common reason for parents’ refusal of vaccinating their children was that they thought their children were not at risk of getting COVID-19 infection (94.1%).

**Figure 4:**
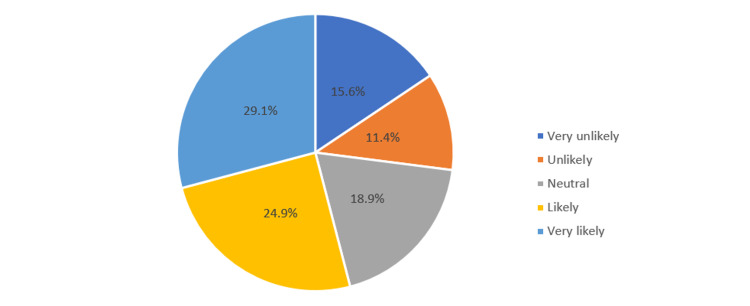
Parents’ acceptability for a child under the age of 18 years to take up free COVID-19 vaccination.

In Figure 4, 53.7% of parents agreed that they were likely allow their children who are below 18 years to take free COVID-19 vaccination as opposed to 27% who were unlikely to do so. 

Table 2 describes the parents’ perception towards COVID-19 vaccination with 3-point Likert scale categories designated as “disagree”, “neutral”, and “agree”. Following the results, we observed that in the positive attitude towards COVID-19 vaccination, 46.5% agreed that COVID-19 vaccination is highly effective in protecting their children from COVID-19, 72.4% agreed that taking up COVID-19 vaccination can contribute to the control of COVID-19 in Saudi Arabia and 86.8% believe that Saudi Arabia will have an adequate supply of COVID-19 vaccination. Based on the above three statements, the overall mean score was 7.75 (SD 1.38). In the negative attitudes towards COVID-19 vaccination, 19.8% believe that COVID-19 vaccination will have severe side effects after receiving the vaccine, 31.5% believe that the protection of COVID-19 vaccines will only last for a short time while 42.3% agreed that their child is afraid of vaccination and 11.1% indicated that lack of time was the main reason for not taking their child for COVID-19 vaccination. The overall mean score of negative attitudes towards COVID-19 vaccination was 7.48 (SD 1.89). Furthermore, nearly half (47.7%) agreed that their family members would support them in taking COVID19 vaccination for their child whereas 74.5% agreed that it would be easy for them to convince their child to take up COVID-19 vaccination. 

**Table 2:** : Parents perception towards COVID-19 vaccination (n=333).

Statement	Agree N (%)
Positive attitude towards COVID-19 vaccination	
Positive attitude scale score (mean ± SD)	7.75 ± 1.38
COVID-19 vaccination is highly effective in protecting your child from COVID-19	155 (46.5%)
Taking up COVID-19 vaccination can contribute to the control of COVID-19 in Saudi Arabia	241 (72.4%)
Saudi Arabia will have an adequate supply of COVID-19 vaccination	289 (86.8%)
Negative attitudes towards COVID-19 vaccination	
Negative attitude scale score (mean ± SD)	7.48 ± 1.89
Your child will have severe side effects after receiving COVID- 19 vaccination	66 (19.8%)
The protection of COVID-19 vaccines will only last for a short time	105 (31.5%)
Your child is afraid of vaccination	141 (42.3%)
You do not have time to take your child for COVID-19 vaccination	37 (11.1%)
Perceived subjective norm related to child’s COVID-19 vaccination: your family members would support you in having your child take up COVID-19 vaccination	159 (47.7%)
Response score (mean ± SD)	2.24 ± 0.81
Perceived behavioral control to have the child take up COVID-19 vaccination: having the child receive COVID-19 vaccination is easy for you if you want them to	248 (74.5%)
Response score (mean ± SD)	2.64 ± 0.66

In Table 3, 47.1% and 21%, respectively, are sometimes or always exposed to positive information related to COVID-19 on social media while 40.5% and 20.7%, respectively, expressed that they were sometimes or always exposed to negative information related to COVID-19 information on social media. Likewise, 29.7% and 14.7%, respectively, stated that they were sometimes or always exposed to testimonials given by participants of the COVID-19 vaccine clinical trials on social media, and 33.6% and 11.7%, respectively, indicated that they were sometimes or always exposed to negative information about other vaccine incidents in Saudi Arabia on social media. 

**Table 3:** Influence of social media related to COVID-19 vaccination.

Statement	N (%)
Frequency of exposure to positive information related to COVID-19 vaccination (eg, new vaccines entering clinical trials, promising efficacies of the vaccines, and vaccines will enter the market soon) on social media	
Response score (mean ± SD)	1.78 ± 0.90
Almost never	37 (11.1%)
Seldom	69 (20.7%)
Sometimes	157 (47.1%)
Always	70 (21.0%)
Frequency of exposure to negative information related to COVID-19 vaccination (eg, concerns about efficacies and supplies, side effects of the vaccines, and receiving vaccines will cause COVID-19) on social media	
Response score (mean ± SD)	1.68 ± 0.96
Almost never	48 (14.4%)
Seldom	81 (24.3%)
Sometimes	135 (40.5%)
Always	69 (20.7%)
Frequency of exposure to testimonials given by participants of the COVID-19 vaccine clinical trials on social media	
Response score (mean ± SD)	1.33 ± 1.02
Almost never	86 (25.8%)
Seldom	99 (29.7%)
Sometimes	99 (29.7%)
Always	49 (14.7%)
Frequency of exposure to negative information about other vaccine incidents in Saudi Arabia (eg, selling problematic vaccines and severe side effects) on social media	
Response score (mean ± SD)	1.34 ± 0.96
Almost never	78 (23.4%)
Seldom	104 (31.2%)
Sometimes	112 (33.6%)
Always	39 (11.7%)

When measuring the relationship between the parental acceptability of a free COVID-19 vaccination and the socio-demographic characteristics of the parents, it was found that age group, history of seasonal influenza vaccination, child age, and avoiding social/meal gathering with other people showed a significant relationship with acceptability to take up child-free COVID-19 vaccine (p<0.05) (see Table 4). 

**Table 4:** Relationship between acceptability and socio-demographic characteristics of the parents (n=333).

Factor	Crude odds ratio (95% CI)	p-value
Age group		
18 – 30 years	Ref	
31 – 40 years	1.915 (1.053 – 3.483)	0.033
>40 years	1.663 (0.961 – 2.877)	0.069
Gender		
Male	Ref	
Female	0.965 (0.627 – 1.486)	0.873
Marital status		
Married	Ref	
Divorced	1.140 (0.387 – 3.359)	0.813
Educational level		
High school or below	Ref	
College degree or higher	0.877 (0.561 – 1.373)	0.567
Type of work		
Frontline worker	Ref	
Management staff	0.770 (0.448 – 1.325)	0.346
Not working	0.651 (0.376 – 1.127)	0.126
History of seasonal influenza vaccination		
No	Ref	
Yes	1.947 (1.258 – 3.014)	0.003
Family history of COVID-19		
No	Ref	
Yes	0.972 (0.631 – 1.496)	0.896
Child age		
0 – 3 years	Ref	
4 – 6 years	3.125 (1.520 – 6.427)	0.002
7 – 12 years	3.571 (1.721 – 7.410)	0.001
13 – 17 years	1.358 (0.713 – 2.587)	0.352
Avoid social/meal gathering with other people who do not live together		
No	Ref	
Yes	1.844 (1.144 – 2.974)	0.012
Avoid crowded places		
No	Ref	
Yes	1.269 (0.705 – 2.285)	0.427

In the multivariate regression model, the positive attitude scale was associated with lower parental acceptability rates (AOR=0.432; 95% CI=0.324-0.570; p<0.001). On the other hand, the negative attitude scale was more associated with higher parental acceptability rates (AOR=1.358; 95% CI=1.158-1.590; p<0.001), while the support of family members in having a child take up COVID-19 vaccination was significantly associated with lower rates of parental acceptability for a free COVID-19 vaccination (AOR=0.403; 95% CI=0.272-0.596; p<0.001). On the other hand, the influence of social media related to COVID-19 vaccination did not show a significant effect on the parental acceptability rates for a free COVID-19 vaccination after adjustment to the regression model (p>0.05) (see Table 5).

**Table 5:** Factors associated with parental acceptability of a free COVID-19 vaccination (n=333).

Factor	AOR (95% CI)	p-value
Perceptions related to COVID-19 vaccination based on the theory of planned behavior		
Positive attitude scale	0.432 (0.327 – 0.570)	<0.001
Negative attitude scale	1.358 (1.158 – 1.590)	<0.001
Your family members would support you in having your child take up COVID-19 vaccination	0.403 (0.272 – 0.596)	<0.001
Having the child receive COVID-19 vaccination is easy for you if you want them to	0.860 (0.536 – 1.379)	0.531
Influence of social media related to COVID-19 vaccination		
Frequency of exposure to positive information related to COVID-19 vaccination on social media	0.939 (0.662 – 1.331)	0.722
Frequency of exposure to negative information related to COVID-19 vaccination on social media	1.307 (0.943 – 1.811)	0.108
Frequency of exposure to testimonials given by participants of the COVID-19 vaccine clinical trials on social media	0.833 (0.617 – 1.124)	0.232
Frequency of exposure to negative information about other vaccine incidents in China on social media	0.850 (0.600 – 1.204)	0.850

## Discussion

As of August 2021, there are many vaccines being distributed and used worldwide among adults and children [8]. However, some people are hesitant to take the vaccine due to rumors spreading in social media and the internet about the potential side effects of the vaccine even though these claims have not been proven scientifically [9]. Unvaccinated parents or children might be susceptible to being infected with COVID-19. Therefore, it is important to explore the factors associated with parental acceptability or refusal for COVID-19 vaccine.

Our study showed that parents who are between 31 and 40 years of age were significantly more likely to accept vaccinating their children against COVID-19. We found that nearly two-thirds (63%) of participants aged 31-40 were college degree holders or higher. Also, parents within this age group had children who are aged more than three years or had more than one child. This is because, in our study, parents who had children older than three years were significantly more likely to accept COVID-19 vaccine for their children. This finding is similar to the study done by Bell et al, which showed high acceptability rate of the COVID-19 vaccination among participants with the main reason being to protect themselves, their children and others. [10]

Other demographic data including educational level and gender does not seem to have a statistical significance on parental acceptability of COVID-19 vaccine to their children. Upon interviewing the parents for possible reasons not accepting COVID-19 vaccine, the majority reported a lack of information and evidence as the main reason. Our findings are in accordance with the study by Bell et al. [10] which showed that lack of evidence was the most commonly reported reason for not accepting COVID-19 vaccine even though the majority were willing to take COVID-19 vaccine both for themselves and their children [10].

In our study, we found that parents who have had a history of taking the seasonal influenza vaccine were significantly more likely to accept vaccinating their children. However, compared to the study done by Zhang et al., [11] there were no significant correlation between taking seasonal vaccine and acceptance of COVID-19 vaccine. The difference in our study and the Chinese study could be attributable to the difference in total COVID-19 versus influenza vaccine acceptance rate. Twenty percent of the participants in the Chinese study took the influenza vaccine as opposed to 72% taking COVID-19 vaccine. Conversely, 50% of our participants took the influenza vaccine at least once leading to 53.7% accepting COVID-19 vaccination.

Parents of children who were willing to accept COVID-19 vaccination to their children were more likely to practice self-precautions against COVID-19. It can be observed that 84.2% and 70.9%, respectively, were avoiding crowded places and social/meal gatherings with other people. This emphasizes the role and effectiveness of spreading awareness by the World Health Organization on different platforms as evident in different studies [3,12,13,14].

The study of social media effect on the prospective and concerns of parents and what they may think regarding a new pandemic or health concern in general is crucial to identify different variables that may affect parents’ level of acceptability to the new COVID-19 vaccine [15,16]. We asked our participants about the frequency of exposure to either negative or positive information regarding COVID-19 and its vaccines. We have found that the influence of social media related to COVID-19 vaccination did not show a significant effect on the parental acceptability rates of COVID-19 vaccination after adjustment to the regression model (p>0.05). Our finding contrasts with a study done by Wang et al., [17] Which showed that social media exposure was associated with a higher parental acceptability rate of COVID-19 vaccine for their children.

Another significant factor in the perception and acceptance of COVID-19 vaccine among the parents was children age groups. The largest children age group was 4-12 accounting for 60.6% of study subjects. The study showed that a child’s age had a statistically significant effect on the acceptability of COVID-19 vaccination among the parents. This finding was similar to what have been found in Bell et al., [11] study, that parents were more likely to vaccinate their children due to the advantages that the vaccine has against the COVID-19 virus. The statistical significance lied between ages 4-12 years. This means that parents who had a child aged between 4-12 years were more likely to accept the vaccine more than those who had a child aged <4 or >12 years. The reason for this positive perception towards COVID-19 vaccine was stated by the parents as we interviewed them. The majority believed that the vaccine will be effective in protecting their children but were afraid of the side effects on very young children, especially those aged three years or younger, since the parents believe that they are most vulnerable. For children between the ages of 13-18, parents were afraid of the vaccines’ effect on pubertal teens and fertility issues among young females. This is especially impacting as these perceptions were disproved by the Saudi Ministry of Health recently [1,2,10].

Another significant factor in determining parents' acceptance of the COVID-19 vaccine was the availability of the vaccine. Surprisingly, we found that parents who believe that COVID-19 vaccine is and will be available were less likely to vaccinate their children. Some of those parents reported, during our interview with them, that because of the ‘huge’ vaccine supply that the Saudi Ministry of Health has, there is no rush to vaccinate their children in the meantime until further studies about the vaccine safety and efficacy is published. Our finding contrasts with other studies that do not mention a significant correlation between parents' acceptability of the vaccine with the availability [7,10,11,18].

Despite these findings, our study has its own limitations. Firstly, This study was conducted at one center in Riyadh, Saudi Arabia. Thus, these findings may not present parents' view regarding COVID-19 vaccine across Saudi Arabia. Secondly, the study used a convenience sampling method followed by an interview of parents who consented to the interview to clarify on their perceptions/answers. We were not able to conduct a full interview to elaborate on the subjects' perception due to subjects' feeling uncomfortable to continue the interview. Thirdly, our sample size was set to be 400 even though this is not a large sample size to find clear statistical significance. Finally, our response rate aim was originally 85%, but we could not reach this rate despite giving more time to data collection.

## Conclusions

As COVID-19 spread globally and made people's lives in danger, vaccination became highly important measure to halt the spread of the disease. Parents are now given the choice of protecting their beloved children from COVID-19 infection and its possible complications. Based on our findings, we noticed that majority of parents plan to vaccinate their children. In addition, certain age groups of parents and children were significantly associated with decreased vaccine hesitancy.
